# Incidence of anaphylaxis and accidental peanut exposure: A systematic review

**DOI:** 10.1002/clt2.12064

**Published:** 2021-10-06

**Authors:** Antonella Muraro, J. Wesley Sublett, Tmirah Haselkorn, Caroline Nilsson, Thomas B. Casale

**Affiliations:** ^1^ Department of Woman and Child Health Food Allergy Referral Centre Veneto Region Padua University Hospital Padua Italy; ^2^ Family Allergy & Asthma Louisville Kentucky USA; ^3^ EpiMetrix, Inc. Los Altos California USA; ^4^ Clinical Science and Education Karolinska Institutet Sodersjukhuset Sachs' Children and Youth Hospital Stockholm Sweden; ^5^ Food Allergy Research and Education (FARE) University of South Florida Tampa Florida USA

**Keywords:** accidental exposure, anaphylaxis, food allergy, peanut allergy, pediatric, allergia al cibo, allergia alle arachidi, anafilassi, esposizione accidentale, pediatrica

## Abstract

**Background:**

Peanut allergy (PA), a common food allergy, is increasing in prevalence and is associated with high rates of anaphylaxis. Prevalence of food‐related anaphylaxis is higher in children and adolescents than in adults, and the pediatric incidence is increasing. We conducted a systematic literature review and meta‐analysis to determine the incidence of peanut‐induced anaphylaxis in children and/or adolescents with PA.

**Methods:**

Literature searches were conducted using the PubMed database and through supplemental methods. Eligible articles for inclusion were peer‐reviewed studies published in English that reported the incidence of anaphylaxis in pediatric PA using the 2006 National Institute of Allergy and Infectious Disease/Food Allergy and Anaphylaxis Network criteria, sample size, and follow‐up duration. Incidence rates were calculated as person‐years at risk or a crude incidence rate was calculated. Meta‐analyses of pooled data were conducted using the *I* ^2^ statistic as the measure of heterogeneity.

**Results:**

A total of 830 citations were screened; 8 met the study inclusion criteria and were selected for review. Pooled meta‐analysis estimates of the incidence of (1) anaphylaxis among children/adolescents with food allergies, (2) anaphylaxis among children/adolescents with PA, and (3) accidental exposure to peanuts among children/adolescents with PA were 3.72 cases per 100 person‐years (95% confidence interval [CI] = 2.35, 5.10), 2.74 cases per 100 person‐years (95% CI = 1.42, 4.05), and 12.28 cases per 100 person‐years (95% CI = 11.51, 13.05), respectively.

**Conclusions:**

The risks of anaphylaxis among children with food allergies and those with PA contribute to the serious overall burden of PA and food allergy for children and their families.

## 1 INTRODUCTION

Peanut allergy (PA) is among the most common food allergies, and is often a lifelong condition associated with risks of severe and potentially fatal allergic reactions, including anaphylaxis, due to accidental peanut exposures.^1^, ^2^, ^3^, ^4^ The general population prevalence of PA in Western countries is estimated to be 1%–2%,^1^, ^5^, ^6^, ^7^, ^8^, ^9^ and some data indicate that it has increased over the past 2 decades.^9^, ^10^, ^11^, ^12^ The prevalence of PA is generally lower in other parts of the world,^9^ although substantial knowledge gaps in this area exist for developing and emerging countries.^13^ Standard management of PA, and other food allergies, consists of strict dietary avoidance of the triggering food and use of intramuscular epinephrine (adrenaline) in cases of allergic reactions due to accidental exposure.^14^, ^15^ Health‐related quality of life is significantly impaired in people with PA and other food allergies and their caregivers due to the constant vigilance required to avoid accidental exposures to trigger foods and associated stress, anxiety, and dietary and lifestyle restrictions.^16^, ^17^, ^18^, ^19^, ^20^

Among the food allergies, PA has been associated in multiple studies with the highest rates of severe reactions, anaphylaxis, and fatal anaphylaxis in Western nations.^21^, ^22^, ^23^, ^24^, ^25^, ^26^, ^27^, ^28^ The largest longitudinal study to date of the rate of accidental peanut exposures in children with PA (*n* = 1941), conducted in Canada, reported an annual incidence of 12.4%, and found that two‐thirds of the exposures caused moderate or severe reactions.^29^ Although fatal food‐related anaphylaxis is rare, with an estimated incidence rate of 1.8 per million person‐years overall, and 3.25 per million person‐years in children, the ongoing and highly unpredictable risk of severe reactions due to accidental exposure contributes greatly to the burden of PA and other food allergies.^19^, ^30^, ^31^, ^32^ In addition, studies in Western nations indicate that rates of food‐related anaphylaxis, including fatal events and hospitalizations, are higher in children and adolescents than in older age groups,^32^, ^33^, ^34^, ^35^ and have been increasing markedly in pediatric age groups.^33^, ^34^, ^35^, ^36^, ^37^, ^38^, ^39^, ^40^, ^41^

Determining the rate of anaphylaxis in the high‐risk population of children and adolescents with PA is important to assess the burden of this risk, and to provide essential data for future analyses of PA‐associated needs for healthcare and auxiliary services and support. While many studies have assessed rates of anaphylaxis in the general population, or in patients with food allergies, specific data for the PA pediatric population are limited. Obtaining such data is particularly important to better understand the effect this information has on clinical decision‐making.

When food allergy is diagnosed, the first‐line treatment has traditionally been avoidance and prescription of rescue medications in the event of accidental exposures. In January 2020, the first treatment for PA was approved by the US Food and Drug Administration, although other immunotherapies using food for PA have been used in phase 2 trials.^42^, ^43^, ^44^, ^45^ In addition to the approved product, oral immunotherapy with foods (not approved by any regulatory authorities) for various food allergies is already being offered and the benefit–risk ratio of this treatment is an important subject for analysis.^46^ In addition, an epicutaneous immunotherapy for PA is in development,^47^ and a range of other potential therapeutic approaches to PA, such as probiotics, biologics, and DNA vaccines, are under investigation.^48^

The objective of this systematic literature review and analysis is to assess the incidence of peanut‐induced anaphylaxis in children and adolescents with PA based on peer‐reviewed published data.

## 2 METHODS

### 2.1 Study eligibility criteria and search strategy

Incidence rate was selected to assess the frequency of anaphylaxis in the pediatric population, as it estimates the rate of new events occurring on a population level during a defined period of time. Anaphylaxis is a severe, potentially life‐threatening systemic hypersensitivity reaction that may or may not recur.^49^ Therefore, we considered anaphylaxis incidence rates to be more informative for practical considerations, such as contribution to PA burden and healthcare needs, than other commonly used rates such as prevalence or cumulative incidence.

The reporting of our search methods, analysis, and results follow the Preferred Reporting Items for Systematic Reviews and Meta‐Analyses (PRISMA) criteria.^50^ Literature search criteria were developed prior to the search (see Appendix Table S1) and the search was not conducted iteratively. One comprehensive screen was applied within the United States National Library of Medicine/National Institutes of Health PubMed database to identify peer‐reviewed, published studies reporting the incidence of anaphylaxis in children and adolescents with PA. To ensure capture of relevant food allergy studies that may have included peanut allergy and provide a comprehensive overview of anaphylaxis among children or adolescents ages 4–17 with PA, we searched for articles that reported (1) incidence of anaphylaxis among children/adolescents with food allergies; (2) incidence of anaphylaxis among children/adolescents with PA; and (3) incidence of accidental peanut exposure in children/adolescents with PA.

Formal study eligibility/inclusion criteria were (1) print or e‐ publication date from January 1, 2000, through May 15, 2019, with a focus on studies that met the 2006 publication of the Second National Institute of Allergy and Infectious Disease/Food Allergy and Anaphylaxis Network (NIAID/FAAN) symposium guidelines, or the so‐called “Sampson's criteria” (see Appendix Table S2),^51^ for the definition of anaphylaxis or studies in which Sampson's criteria could be applied; (2) peer‐reviewed studies published in English; (3) studies including children or adolescents aged 4–17 years; (4) report of the incidence rate, or number of incident cases, of anaphylaxis; (5) report of the sample size; and (6) report of the average or median length of follow‐up. To identify eligible publications, Medical Subject Headings (MeSH) and non‐MeSH terms were applied in various combinations including (with MeSH terms in parentheses): peanut allergy, (peanut hypersensitivity), (food hypersensitivity), (anaphylaxis), (incidence), (epidemiology), accidental exposure, children, adolescents, pediatric, and (pediatrics). Studies reporting the results from clinical trials and/or oral food challenges were excluded from this review.

### 2.2 Definition of anaphylaxis for this meta‐analysis

Sampson's criteria for anaphylaxis (see Appendix Table S2) was used to define anaphylaxis in each of the studies. In instances where Sampson's criteria were not explicitly used for the definition of anaphylaxis, studies that used one of three definitions of anaphylaxis were included: (1) an explicit description of cases as anaphylaxis; (2) reported allergic reactions by severity (mild, moderate, severe); those categorized as “severe allergic reactions” were considered to be anaphylaxis; (3) reported “anaphylactic reaction.”

### 2.3 Data collection/extraction

Studies were included if incidence rate data specific to the type of allergy (e.g., peanut allergy, all food allergy) were reported. If incidence rates were not directly reported, studies which provided the necessary data for the calculation of incidence rates were included. Incidence rates were defined as the number of incident cases of anaphylaxis divided by the person‐years at risk. In the studies reviewed, incidence rates were typically provided per 1000 person‐years. In instances where a study did not explicitly report the incidence rate, but reported the number of incident cases of anaphylaxis, the average length of follow‐up, and the sample size, a crude incidence rate was calculated as follows:

$$Incidence\;rate=\frac{\#\;of\;new\;anaphylaxis\;cases}{(average\;length\;of\;follow‐up)\;\times \;sample\;size}$$

If the article did not provide *average* length of follow‐up but provided *median* length of follow‐up time, a crude incidence rate was calculated as follows:

$$Incidence\;rate=\frac{\#\;of\;new\;anaphylaxis\;cases}{(median\;length\;of\;follow‐up)\times sample\;size}$$

A minority of the included studies provided 95% confidence intervals (CIs) with their point estimates. The following formulas^52^ were used to calculate crude 95% CI for the studies that did not report 95% CI:

r=incidencerate

$$\mathrm{Standard}\;\mathrm{Error}=\;\sqrt{\left(\frac{1-r}{\#\;incident\;cases}\right)}$$

$$95\%\;CI=\left(e^{\ln\;r-z_{1-\frac{0.05}{2}}SE},e^{\ln\;r+z_{1-\frac{0.05}{2}}SE}\right)$$

### 2.4 Meta‐analysis

Pooled incidence rates were calculated among three populations of interest (children/adolescents with PA, with food allergy, and with PA who experienced accidental exposure). The pooling was performed using a random effects model.^53^ Heterogeneity is presented as the *I* ^2^ statistic, which describes the percentage of variation across studies that is due to heterogeneity rather than chance.^54^ As observed with the chi‐squared statistic, the *p*‐value associated with the *I* ^2^ statistic registers as statistically significant if any one study differs from another.

### 2.5 Publication quality and bias assessment

We used the Q‐Coh scale to assess the quality of individual studies and their risk of bias.^55^ This scale encompasses 26 individual items organized in eight major domains: study design, representativeness, comparability of groups, maintenance of comparability, exposure measure, outcome measure, attrition, and statistical analyses. Studies were deemed to be of “good” quality if at least 6 domain items were scored positively and of “acceptable” quality if 4–5 domain items were scored positively. Studies that were considered of less than “good” or “acceptable” quality were excluded from this review.

## 3 RESULTS

### 3.1 Literature search

**FIGURE 1:**
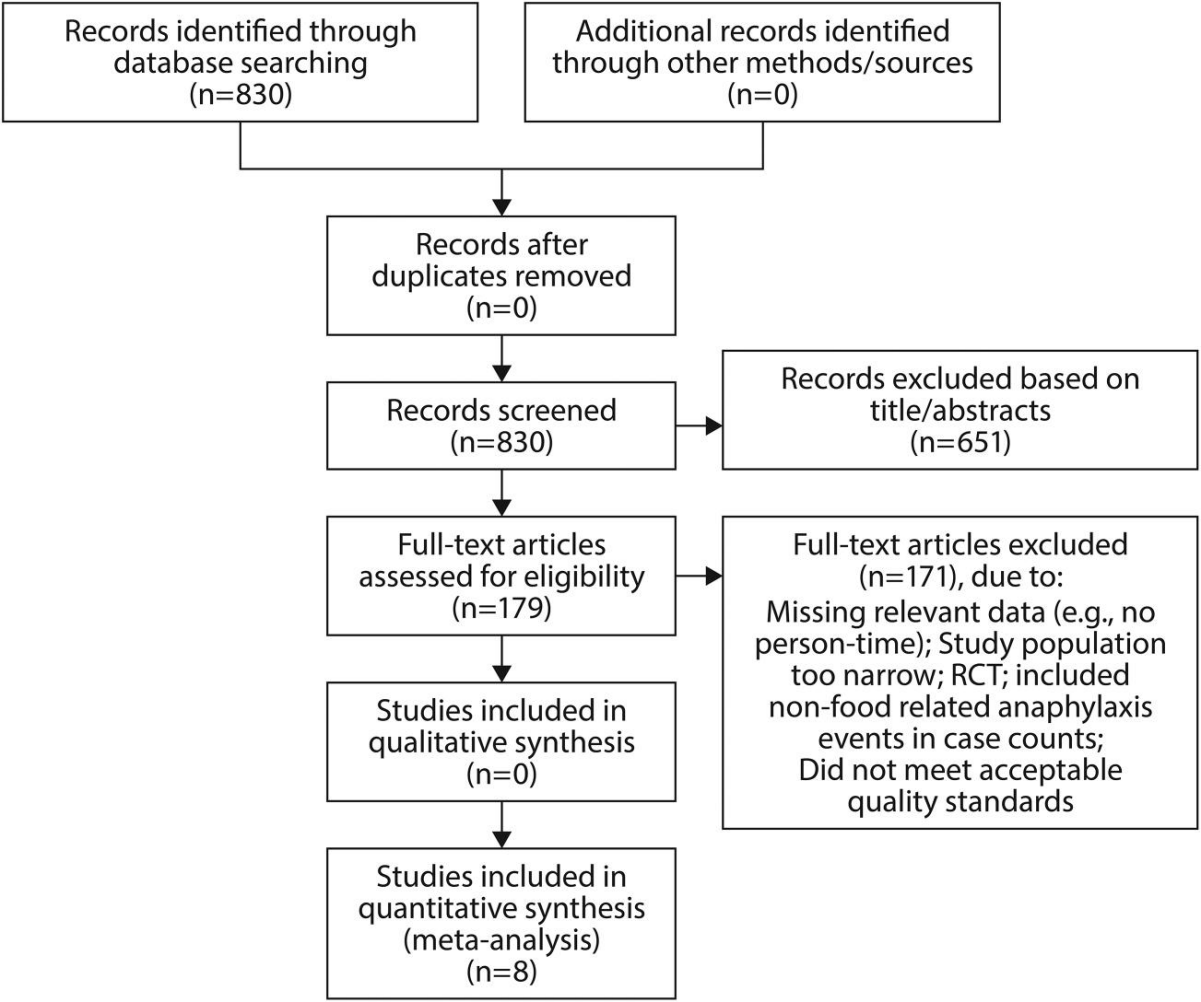
Flowchart showing the results of the search and selection strategy for eligible studies. RCT, randomized controlled trial

As shown in Figure 1, which provides the results of the search strategy described above, the initial database search of PubMed yielded 830 total records. Since the search strategy was intended to be broad and included multiple common search terms, the majority of the records identified were not eligible for inclusion. Screening of titles and abstracts led to exclusion of 822 for failure to meet eligibility criteria (Figure 1). As a result, eight articles were included in the systematic review and meta‐analysis of pooled data. All of these were of “good quality” according to the Q‐Coh scale.

As described in Section 2, three search outputs were collected. Tables 1, 2, 3 summarize the anaphylaxis incidence data captured from one or more studies identified with each output.

**TABLE 1:** Characteristics of studies that report the incidence of anaphylaxis^a^ among children/adolescents with food allergies^b^

Author (year published)	Type of allergy	Sample size	Age range	Number of patients who experienced anaphylaxis	Reported or derived person‐years	Reported or derived incidence rate (95% CI; per 100 person‐years)
Boyano‐Martinez et al. (2009)^56^	Cow milk	88	18–147 months	6	85	7.06 (3.26, 15.27)
Clark and Ewan (2008)^57^	Tree nut or peanut	785	40–107 months	45	3640	1.23 (0.92, 1.65)
Fleischer et al. (2012)^47^	Milk or egg	512	3–15 months	134	1514.7	8.85 (7.53, 10.40)
Vander Leek et al. (2000)^58^	Peanut	83	0.4–6.8 years	41	489.7	8.37 (6.24, 11.22)
Yu et al. (2006)^59^	Peanut	252	4–17 years	4	244	1.64 (0.62, 4.33)
Nguyen‐Luu et al. (2012)^60^	Peanut	1411	0–17 years	43	2227	1.93 (1.44, 2.60)
Neuman‐Sunshine et al. (2012)^61^	Peanut	782	0–16 years	71	4526	1.57 (1.25, 1.98)

Abbreviation: CI, confidence interval.

^a^ Sampson's criteria for anaphylaxis (see Appendix Table S2) were used to define anaphylaxis in each of the studies. In instances where Sampson's criteria were not explicitly used for the definition of anaphylaxis, studies that used one of three definitions of anaphylaxis were included: (1) an explicit description of cases as anaphylaxis; (2) reported allergic reactions by severity (mild, moderate, severe); those categorized as “severe allergic reactions” were considered to be anaphylaxis; (3) reported “anaphylactic reaction.”

^b^ Studies that included children or adolescents aged 4‐17 years and reported the incidence rate, or number of incident cases, of anaphylaxis, sample size, and the average or median length of follow‐up were included in the meta‐analysis.

**TABLE 2:** Characteristics of studies that report the incidence of anaphylaxis among children/adolescents with peanut allergies

Author (year published)	Sample size	Number of patients who experienced anaphylaxis	Reported or derived person‐years	Reported or derived incidence rate (95% CI; per 100 person‐years)
Yu (2006)^59^	252	4	244	1.64 (0.62, 4.33)
Vander Leek (2000)^58^	83	41	489.7	8.37 (6.24, 11.22)
Nguyen‐Luu (2012)^60^	1411	43	2227	1.93 (1.44, 2.60)
Neuman‐Sunshine (2012)^61^	782	71	4526	1.57 (1.25, 1.98)

Abbreviation: CI, confidence interval.

**TABLE 3:** Characteristics of studies that report the incidence of accidental exposure among children/adolescents with peanut allergies

Author (year published)	Sample size	Number accidental exposures	Reported or derived person‐years	Reported or derived incidence rate (95% CI; per 100 person‐years)
Nguyen‐Luu et al. (2012)^60^	1411	266	2227	11.94 (10.67, 13.37)
Yu et al. (2006)^59^	252	35	244	14.34 (10.56, 19.49)
Cherkaoui et al. (2015)^29^	1941	567	4589	12.36 (11.44, 13.35)

Abbreviation: CI, confidence interval.

#### 3.1.1 Search Output 1. Incidence of anaphylaxis among children/adolescents with food allergies

The incidence of anaphylaxis among children/adolescents with food allergies was either reported in or derived from seven studies conducted in Canada, Spain, the United Kingdom, or the United States. Table 1 describes the key characteristics of these studies.

Boyano‐Martinez et al. (2009) reported findings from a 12‐month observational study of Spanish children allergic to cow's milk.^56^ Six children experienced severe allergic reactions over approximately 85 person‐years. Clark and Ewan (2008) reported results from a prospective study of 785 children in the United Kingdom with nut allergies.^57^ Participants were recruited from Allergy Centre in Addenbrooke's Hospital, Cambridge. Forty‐five patients experienced a severe allergic reaction over 3640 person‐years of follow‐up. Fleischer et al. (2012) reported findings from a five‐site observational study in the United States (New York, NY; Baltimore, MD; Little Rock, AR; Denver, CO; and Durham, NC) of 512 infants with allergy to milk or egg.^62^ In total, 134 infants experienced severe allergic reactions over 1514.7 person‐years of follow‐up. Vander Leek et al. (2000) reported findings from an observational cohort study of 83 children with diagnosed clinical peanut hypersensitivity who were contacted yearly to track allergic reactions to peanuts.^58^ Allergic reactions were categorized as “non‐life‐threatening” or “potentially life‐threatening”; for this review, those classified in the latter category were considered as anaphylactic reactions. In total, 41 participants experienced an anaphylactic reaction. Yu et al. (2006) reported findings from a cohort study of children with PA diagnosed at the Montreal Children's Hospital.^59^ Of the 252 children, four experienced severe allergic reactions that would be considered anaphylactic reactions over 244 person‐years. Nguyen‐Luu et al. (2006) reported findings from a study of Canadian children with PA recruited from the Allergy Clinics at the Montreal Children's Hospital, provincial and national advocacy organizations for food‐allergic patients (Anaphylaxis Canada, Association Québécoise des Allergies Alimentaires, and the Allergy/Asthma Information Association), and organizations providing products to persons with allergies.^60^ Of the 1411 children recruited, 43 experienced allergic reactions over 2227 person‐years. Neuman‐Sunshine et al. (2012) reported results from a cohort study of 782 children/adolescents in the United States who were diagnosed with PA.^61^ Seventy‐one anaphylactic reactions were reported over approximately 4526 person‐years.

**FIGURE 2:**
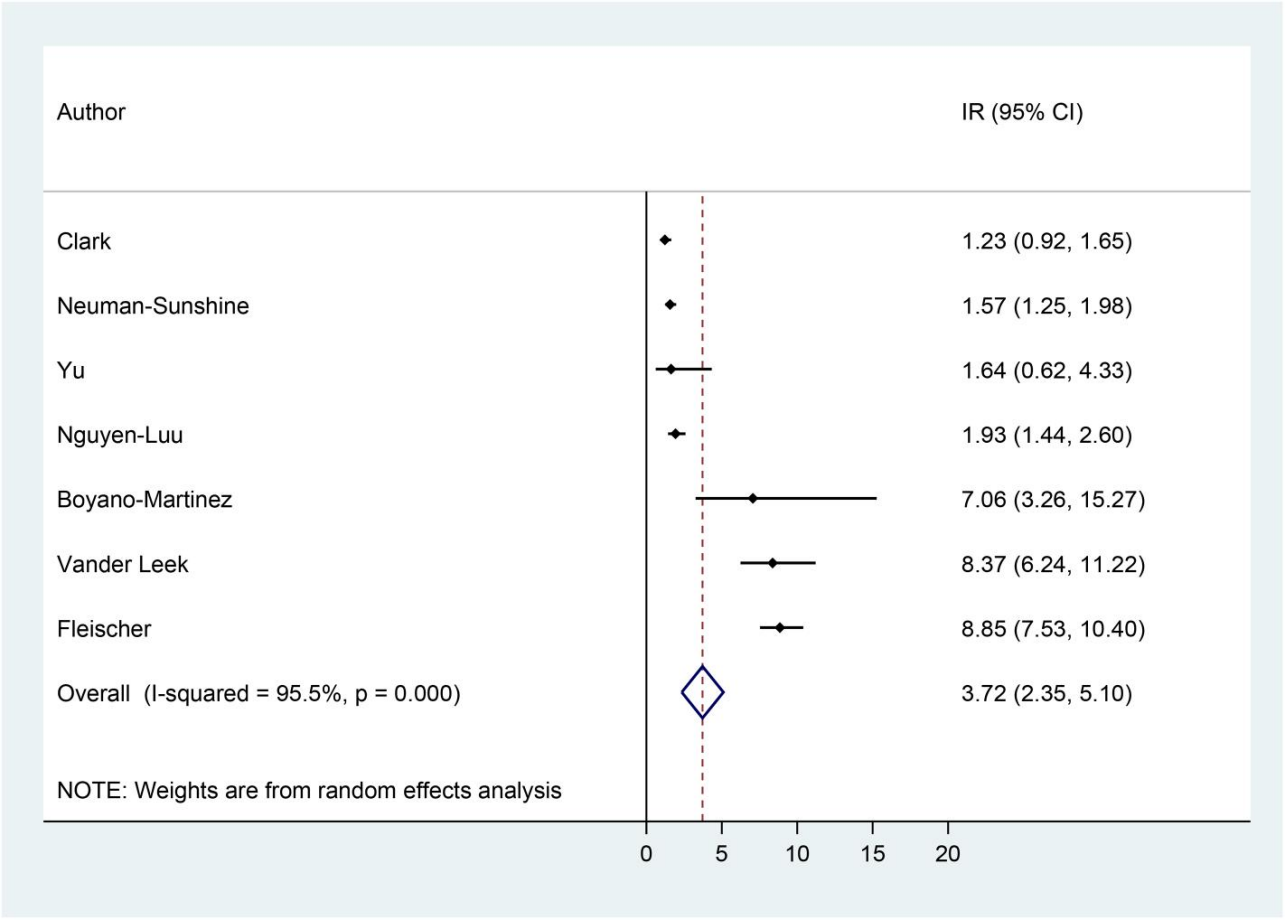
Incidence rate of anaphylaxis among children/adolescents with food allergies.^56^, ^57^, ^58^, ^59^, ^60^, ^61^, ^62^ CI, confidence interval; IR, incidence rate

Incidence rates ranged from 1.23 incident cases of anaphylaxis per 100 person‐years^57^ to 8.85 cases per 100 person‐years.^62^ Based on the meta‐analysis, the pooled estimate of the incidence rate of anaphylaxis among children/adolescents with food allergies was 3.72 incident cases of anaphylaxis per 100 person‐years (95% CI = 2.35, 5.10), with 95.5% heterogeneity (Figure 2).

#### 3.1.2 Search Output 2. Incidence of anaphylaxis among children/adolescents with peanut allergy

Of the eight studies included in Search Output 1, four studies were conducted exclusively among children with PA. Table 2 describes the key characteristics of these studies.

**FIGURE 3:**
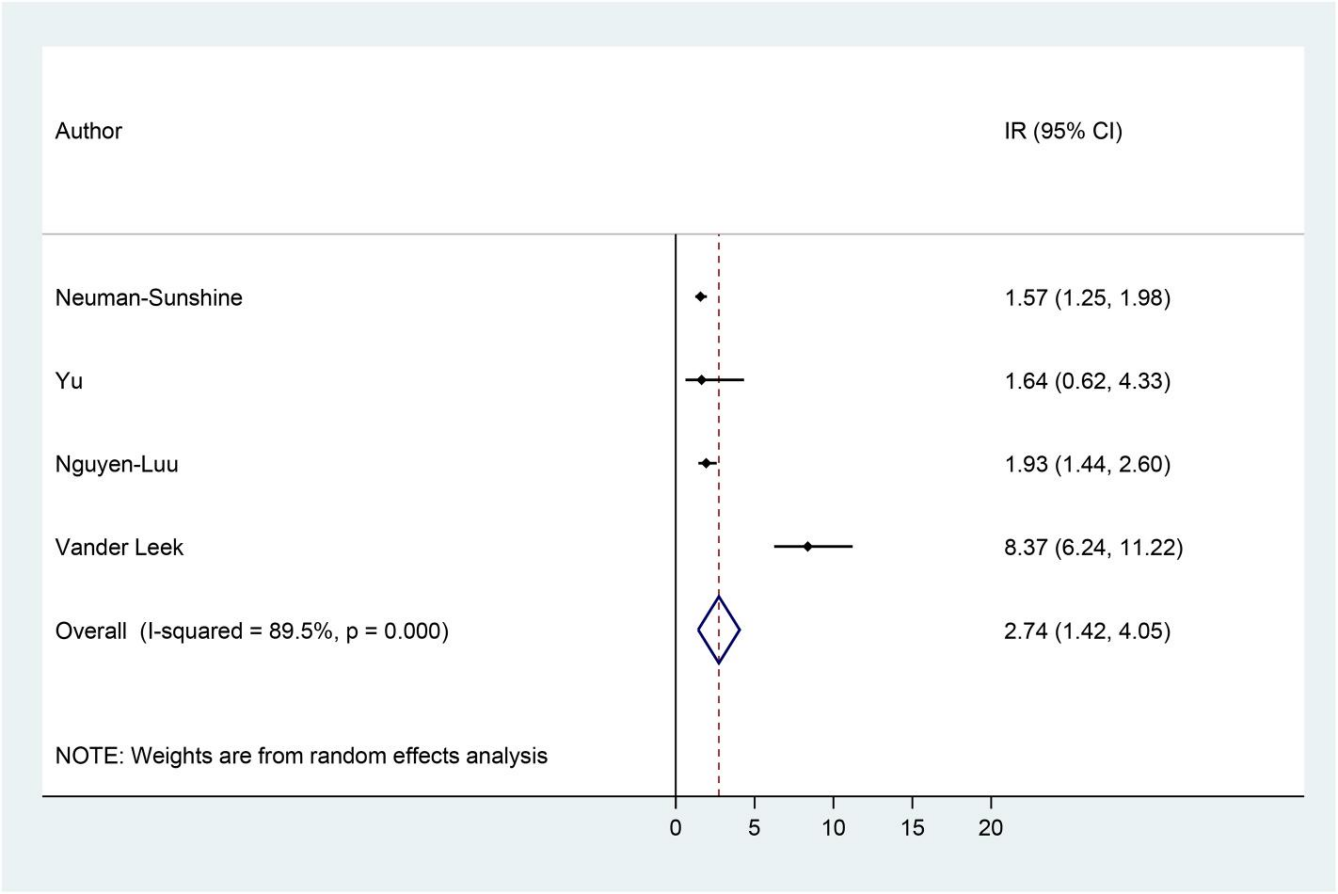
Incidence rate of anaphylaxis among children/adolescents with peanut allergy.^58^, ^59^, ^60^, ^61^ CI, confidence interval; IR, incidence rate

Incidence rates ranged from 1.57 incident cases of anaphylaxis per 100 person‐years^61^ to 8.37 cases per 100 person‐years.^58^ Based on the meta‐analysis, the pooled estimate of the incidence rate of anaphylaxis among children/adolescents with food allergies is 2.74 incident cases of anaphylaxis per 100 person‐years (95% CI = 1.42, 4.05), with 89.5% heterogeneity (Figure 3).

#### 3.1.3 Search Output 3. Incidence of accidental exposure to peanuts among children/adolescents with peanut allergy

The incidence rate of accidental exposure to peanuts among children/adolescents with PA was either reported in or derived for the three studies described in Table 3.

**FIGURE 4:**
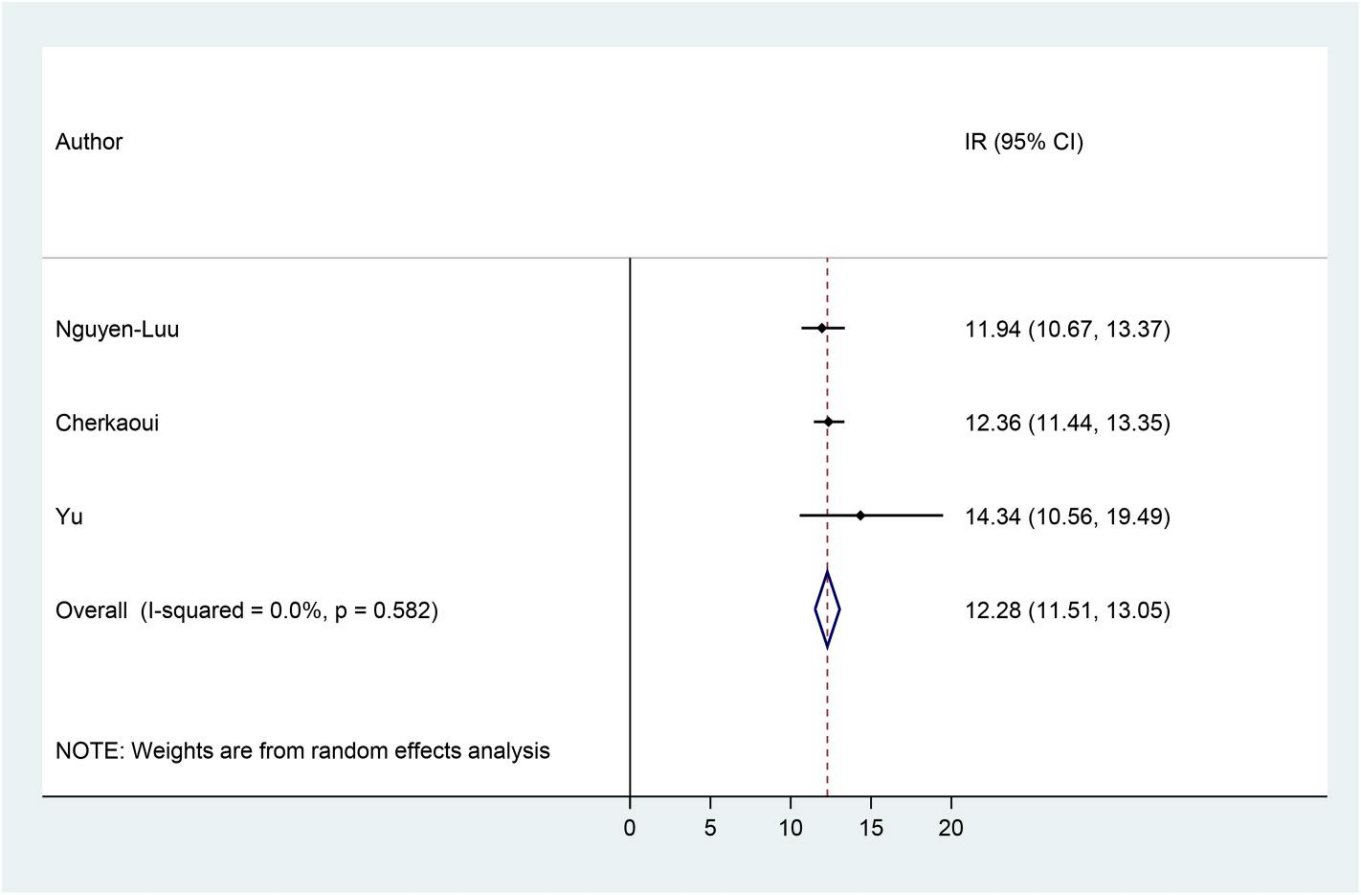
Incidence rate of accidental peanut exposure among children/adolescents with peanut allergy.^29^, ^59^, ^60^ CI, confidence interval; IR, incidence rate

Incidence rates ranged from 11.94 accidental exposures per 100 person‐years^60^ to 14.34 per 100 person‐years.^59^ The pooled meta‐analysis of the incidence rate of accidental exposures to peanuts among children/adolescents with PA is 12.28 cases per 100 person‐years (95% CI = 11.51, 13.05), with 0.0% heterogeneity (Figure 4).

## 4 DISCUSSION

Anaphylaxis is a distressing and potentially fatal event; people may develop stress disorders and psychiatric comorbidity symptoms after experiencing anaphylaxis, which may then impact the way they and their families cope with food and PA.^63^, ^64^, ^65^ The risk of anaphylaxis is associated with physical, cognitive, and behavioral aspects of anxiety that must be addressed in order to ensure optimal psychological as well as medical outcomes.^63^, ^64^, ^65^ More information regarding anaphylaxis can help the health professional to better support and manage the patient and their caregivers suffering from these events in an evidence‐based and cost‐effective manner.^63^, ^64^, ^65^, ^66^, ^67^

This comprehensive systematic literature review and meta‐analysis summarizes the reported incidence of anaphylaxis in children and adolescents with food allergies, PA, and accidental exposure to PA in the literature following the formulation of guideline‐based standardized definitions. To summarize, the estimated incidence rates of the risks of interest in pediatric populations based on pooled analysis of the available studies were: 3.72 per 100 person‐years (95% CI = 2.35, 5.10) for anaphylaxis in children/adolescents with food allergy (7 studies); 2.74 per 100 person‐years (95% CI = 1.42, 4.05) for anaphylaxis in children/adolescents with PA (4 studies); and 12.28 per 100 person‐years for accidental exposures to peanut for children/adults with PA (95% CI = 11.51, 13.05) (3 studies). Taken together, these findings help form a comprehensive picture of the risks of accidental exposures and severe reactions associated with PA.

To our knowledge, only two other systematic reviews and/or meta‐analyses of anaphylaxis incidence in pediatrics have been published since 2006.^68^, ^69^ Wang et al. (2019) conducted a systematic review of the global incidence of anaphylaxis in children in the general population without any limitations with respect to year of publication noted.^68^ These investigators identified 59 studies providing incidence or prevalence of anaphylaxis in more than 20 countries and across four continents. The study reported a total anaphylaxis (regardless of trigger) incidence range of 1–761 per 100,000 person‐years; individual food‐induced anaphylaxis, including peanut‐related, had an incidence range of 0.1–9.7 per 100,000 person‐years, which was higher than the other triggers studied (insect, anesthesia, and serum). Umasunthar et al. conducted a systematic review and meta‐analysis of the incidence of food‐induced anaphylaxis in people with food allergies, including sub‐analyses in “young people” aged 0–19 years, and in individuals (of any age) with PA.^69^ This analysis found that the incidence rates per 100 person‐years for self‐reported food‐induced anaphylaxis were: 8.59 (95% CI: 8.25, 8.94) for all ages (based on 1 study) and 4.93 (95% CI: 2.78, 8.74; range: 0.60, 57.89) for those aged 0–19 years (based on 10 studies). The sub‐meta‐analysis of data for individuals with PA of any age was based on four studies and showed an anaphylaxis incidence rate of 2.64 (95% CI: 1.13, 6.17; range 1.64, 8.90) per 100 person‐years. Overall, the authors found that the highest rates of medically coded anaphylaxis and hospital admission for food anaphylaxis occurred in preschool children.

The studies included in this meta‐analysis showed a higher range of peanut‐induced anaphylaxis cumulative incidence in preschool and older children (14.7%–15.5%) than in adolescents (6.9%) with food allergy; the overall range (children and adolescents combined) was approximately 1%–9% (Table 1). Two other studies of note that evaluated incidence of severe allergic reactions in children with PA were reported prior to publication of the 2006 NIAID/FAAN guidelines.^58^, ^70^ Sicherer et al. (1998) conducted a questionnaire survey of 122 children who had experienced at least one “acute reaction” to peanut or tree nuts, defined as “a reaction involving one or more symptoms in one or more of the following organ systems (skin—hives, edema; respiratory—wheezing, throat tightness, repetitive coughing, shortness of breath; or gastrointestinal [GI] tract—vomiting, diarrhea) within 60 min of exposure.” The investigators further stated that such acute reactions are a “potential cause of anaphylaxis and death” although anaphylaxis itself was not defined for this study.^70^ Of the total study sample (*n* = 122), 102 children (83.6%) (median age 8.0 years [range not given]) had experienced an acute reaction to peanut. Three organ systems were affected in 21% of initial reactions. Overall, in children with PA separately, accidental exposures were reported in 55% over a 5.5‐year period, and the acute reaction occurred at a median age of 24 months.

Vander Leek et al. (2000) conducted a longitudinal study in 83 children with clinical PA diagnosed before their fourth birthday and enrolled at a mean age of 2.4 years, with follow‐up of up to 22 years (median follow‐up, 5.9 years [range, 1.4–22.4 years]).^58^ This study also did not investigate anaphylaxis stated as such but did report events that were “potentially life‐threatening—throat tightness and angioedema, angioedema in the mouth, cough, wheeze, chest tightness, shortness of breath, noisy breathing, tachypnea, voice change.” The study found that 22 children (26.5%) had a “potentially life‐threatening reaction” during follow‐up. In addition, 19 of 43 other children (44.2%) whose index reactions were not life‐threatening had subsequent life‐threatening reactions during follow‐up.

### 4.1 Strengths and limitations

Our study helps to confirm and clarify these previously reported data with the use of more consistent and well‐validated criteria for peanut‐induced anaphylaxis and meta‐analysis using person‐year rates of incidence. Among its strengths, this analysis focused on studies that met the criteria for diagnosis of anaphylaxis as defined in the 2006 publication of the NIAID/FAAN, thus reducing the potential volume of data, but theoretically enhancing its consistency and accuracy. Other strengths include our analysis of different incidence rates/populations, including anaphylaxis in children/adolescents with food allergy, anaphylaxis in children/adolescents with PA, and accidental exposure to peanut in children/adolescents with PA, to provide the appropriate context and scope for analysis of the burden of peanut‐induced pediatric anaphylaxis. Limitations of this analysis include the use of only more recent data. However, as current data indicate a rising prevalence of PA (and other food allergies) in Western countries, and in countries in other parts of the world with Western influence, in recent decades, this report provides a more current and accurate estimate of the scope of the problem.^71^, ^72^ Another limitation is the absence of a widely accepted definition of anaphylaxis and the lack of a consistent method for grading an anaphylactic attack or its severity. Although we attempted to focus on a consistent definition of anaphylaxis and used mostly post‐2006 studies, the definitions of anaphylaxis still varied across the literature sample. In addition, current *International Classification of Diseases* codes for anaphylaxis, used in some studies, may not be fully reliable.^23^ This lack of a consistent definition led to heterogeneity between studies. Other limitations of these data include the exclusion of studies with children <4 years of age and the factor that individuals who participate in research studies and registries may differ from the general target population in health awareness, literacy, and behaviors. Use of the mean follow‐up time as performed for this analysis could also result in underestimation of anaphylaxis rates.

## 5 CONCLUSION

The pooled incidence rates of anaphylaxis among children with food allergies and children with PA are approximately 3.72 cases per 100 person‐years and 2.74 cases per 100 person‐years, respectively. The incidence of accidental exposure was 12.28 cases per 100 person‐years. From a clinical perspective, these data help clarify the risks and burden of PA when physicians are counseling their patients and caregivers as these data indicate that the incidence of anaphylaxis events per patient‐year in food allergy is 0.037, in anaphylaxis in PA is 0.027, and in accidental exposure is 0.123, indicating a high disease burden. Additional confirmatory data and analyses on the incidence of anaphylaxis in pediatric PA in the light of the small number of available studies are needed.

## CONFLICT OF INTERESTS

Antonella Muraro reports speaker fees from Aimmune Therapeutics, DBV, and Nestlé Purina; and is an advisory board member/fees from Regeneron IDMC.

Tmirah Haselkorn is a former consultant to Aimmune Therapeutics, Inc.

J. Wesley Sublett reports the following financial disclosures: Aimmune Therapeutics, ALK‐Abelló, Allergy Therapeutics, AstraZeneca, DBV Technologies, GlaxoSmithKline, Kaleo Pharmaceuticals, Leo Pharma, Mylan, Novartis, Pearl Therapeutics, Perrigo, Pfizer, Roche, Sanofi, Stallergenes Greer, Teva Pharmaceuticals.

Caroline Nilsson reports grants to institution and advisory board fees from Aimmune Therapeutics and Novartis; speakers fees from MEDA, ALK, Thermo Fisher, GSK.

Thomas B. Casale reports he is an investigator on studies awarded to his university from Aimmune Therapeutics.

## AUTHOR CONTRIBUTIONS

Antonella Muraro: Conceptualization; equal, investigation; equal, methodology; equal, project administration; equal, resources; equal, supervision; equal, visualization; equal, writing‐original draft; equal, writing‐review and editing; equal. J. Wesley Sublett: Investigation; equal, methodology; equal, resources; equal, visualization; equal, writing‐original draft; equal, writing‐review and editing; equal. Tmirah Haselkorn: Conceptualization; equal, data curation; equal, investigation; equal, methodology; equal, project administration; equal, resources; equal, software; equal, validation; equal, visualization; equal, writing‐original draft; equal, writing‐review and editing; equal. Caroline Nilsson: Investigation; equal, methodology; equal, resources; equal, visualization; equal, writing‐original draft; equal, writing‐review and editing; equal. Thomas B. Casale: Conceptualization; equal, investigation; equal, methodology; equal, project administration; equal, resources; equal, supervision; equal, visualization; equal, writing‐original draft; equal, writing‐review and editing; equal.

## Supporting information

Supporting Information S1
