# Supplementary material for: Incidence of anaphylaxis and accidental peanut exposure: A systematic review
Source: Clin Transl Allergy. 2021 Oct 6;11(8):e12064. doi: 10.1002/clt2.12064 (PMC8694181; doi:10.1002/clt2.12064)
Supplement: Supplementary file 1 — Supporting Information S1 [file CLT2-11-e12064-s001.docx]

**Appendices**

**Table S1. PubMed Search Strategy**

| **Search criteria and terms** |
| --- |
| (incidence or (exposure) or (exposures) or (epidemiology) or (accidental exposure) and ((allergies) or (allergic) or (allergy) or (food allergy) or (food allergies) or (peanut allergy) or (peanut hypersensitivity) or (food hypersensitivity) or (anaphylaxis) or (anaphylactic)) AND (("2000"[Date - Publication] : "2019/04/26"[Date - Publication]))) |
| ((incidence or (exposure) or (exposures) or (epidemiology) or (accidental exposure)) and ((allergies) or (allergic) or (allergy) or (food allergy) or (food allergies) or (peanut allergy) or (peanut hypersensitivity) or (food sensitivity) or (anaphylaxis) or (anaphylactic)) and ((children) or (adolescence) or (adolescents) or (adolescent) or (pediatric) or (pediatrics)) AND (("2000"[Date - Publication] : "2019/04/26"[Date - Publication]))) |

**Table S2.** **Clinical criteria for diagnosing anaphylaxis**

| **Anaphylaxis is highly likely when any one of the following 3 criteria are fulfilled:** |
| --- |
| 1. Acute onset of an illness (minutes to several hours) with involvement of the skin, mucosal tissue, or both (eg, generalized hives, pruritus or flushing, swollen lips-tongue-uvula)   AND AT LEAST ONE OF THE FOLLOWING   1. Respiratory compromise (eg, dyspnea, wheeze-bronchospasm, stridor, reduced PEF, hypoxemia) 2. Reduced BP or associated symptoms of end-organ dysfunction (eg, hypotonia [collapse], syncope, incontinence) |
| 1. Two or more of the following that occur rapidly after exposure to a likely allergen for that patient (minutes to several hours): 2. Involvement of the skin-mucosal tissue (eg, generalized hives, itch-flush, swollen lips-tongue-uvula) 3. Respiratory compromise (eg, dyspnea, wheeze-bronchospasm, stridor, reduced PEF, hypoxemia) 4. Reduced BP or associated symptoms (eg, hypotonia [collapse], syncope, incontinence) 5. Persistent gastrointestinal symptoms (eg, crampy abdominal pain, vomiting) |
| 1. Reduced BP after exposure to known allergen for that patient (minutes to several hours): 2. Infants and children: low systolic BP (age specific) or greater than 30% decrease in systolic BP* 3. Adults: systolic BP of less than 90 mm Hg or greater than 30% decrease from that person’s baseline |

PEF, Peak expiratory flow; BP, blood pressure.

*Low systolic blood pressure for children is defined as less than 70 mm Hg from 1 month to 1 year, less than (70 mm Hg 1 [2 3 age]) from 1 to 10 years, 90 mm Hg from 11 to 17 years.

Reprinted from Second symposium on the definition and management of anaphylaxis: summary report--Second National Institute of Allergy and Infectious Disease/Food Allergy and Anaphylaxis Network symposium, Sampson HA, et al, *J Allergy Clin Immunol* 2006;117:391-7, with permission from Elsevier.
